# Sociodemographic inequalities in the global burden trends and machine learning-based projections of periodontitis from 1990 to 2030 across different development levels

**DOI:** 10.3389/froh.2025.1609961

**Published:** 2025-06-17

**Authors:** Amr Sayed Ghanem, Róbert Bata, Nóra Kovács, Attila Csaba Nagy

**Affiliations:** ^1^ Department of Health Informatics, Faculty of Health Sciences, University of Debrecen, Debrecen, Hungary; ^2^ Department of Public Health and Epidemiology, Faculty of Medicine, University of Debrecen, Debrecen, Hungary

**Keywords:** disease burden, global burden of disease, inequalities, sociodemographic, oral cavity, periodontitis, periodontal disease, joinpoint regression

## Abstract

**Introduction:**

Oral diseases affect billions globally, with periodontitis contributing to significant health disparities and systemic conditions like diabetes and cardiovascular diseases.

**Methods:**

This study utilized the GBD 2021 dataset to assess the global burden of periodontitis across 204 countries, analyzing prevalence, incidence, and DALY rates. Socioeconomic disparities were examined using the Sociodemographic Index (SDI) and Gini coefficient, while time-series analysis, regression models, and Joinpoint regression identified trends. Machine learning predicted future burden, and geospatial mapping visualized global distribution.

**Results:**

Periodontitis burden remains highest in low-SDI regions, with significantly greater prevalence, incidence, and DALY rates compared to higher-SDI countries (*p* < 0.001). Global trends showed a decline until 2010 (AAPC: ASPR −0.792%, ASIR −0.719%, DALY −0.794%; all *p* < 0.05), followed by a temporary increase before stabilizing. Disparities widened over time, peaking around 2010. Projections suggest persistent inequalities, with low-SDI regions maintaining the highest burden and minimal expected reductions, while higher-SDI countries exhibit stable, lower rates.

**Conclusion:**

Despite global declines, periodontitis disparities have widened, with low-SDI regions facing the highest burden and minimal improvements. Without strong public health policies integrating preventive oral health into diseases management, inequalities will persist, worsening systemic health outcomes. Urgent action is needed to ensure universal access to periodontal care and early interventions, especially in low-resource settings.

## 1 Introduction

Oral diseases represent a significant yet often under-recognized global public health challenge, affecting approximately 3.5 billion individuals worldwide, positioning them among the most prevalent health conditions globally (1, 2). These diseases, including dental caries (both untreated deciduous and permanent teeth), severe periodontitis, edentulism, and cancers of the lip and oral cavity, collectively surpass the global disease burden of major non-communicable diseases such as cardiovascular diseases, diabetes mellitus, cancers, chronic respiratory illnesses, and mental disorders combined (1, 2). The greatest burden arises from untreated caries of permanent teeth, with around 2 billion cases globally, followed by severe periodontal disease affecting approximately 1 billion adults, untreated deciduous tooth caries impacting about 510 million individuals, and edentulism observed in 350 million people (3). This burden disproportionately affects lower- and upper-middle-income countries, where three-quarters of the global cases are concentrated, reflecting substantial disparities driven by socioeconomic factors and limited healthcare accessibility (1). Despite variations in population characteristics and healthcare infrastructure across World Bank country income groups and WHO regions, the global average prevalence of oral diseases remains uniformly high at approximately 45%, highlighting systemic gaps in preventive care and oral health services (1). Between 1990 and 2019, the global burden of oral diseases increased significantly, with case numbers rising by approximately 50%, surpassing the global population growth rate of 45% (3). This increase was particularly pronounced in low-income (114%) and lower-middle-income (70%) countries, underscoring an exacerbation of health inequalities (1, 3). Concurrently, disability-adjusted life years (DALYs) attributable to oral conditions rose by 75%, with the steepest increases documented in low-income (123%) and lower-middle-income (98%) nations, reflecting not only an expansion in disease incidence but also a notable rise in conditions associated with higher morbidity, particularly severe periodontal disease (3) which is defined as a chronic inflammatory disease characterized by irreversible damage to the supportive tissues of teeth including gingival tissues, alveolar bone, cementum, and periodontal ligament (4), is a particularly significant public health concern due to its high prevalence and severe clinical consequences. Originating from untreated gingivitis, which affects up to 90% of the global population (5) and is largely reversible through improved oral hygiene, periodontitis emerges when inflammation progresses deeper into periodontal structures, resulting in the destruction of periodontal attachment and subsequent alveolar bone loss (6). This destructive process is driven not only by bacterial invasion but also by the host's inflammatory immune response (4). Ultimately, severe periodontitis can lead to tooth loss, impaired oral function, and reduced quality of life, disproportionately affecting disadvantaged populations who often face greater barriers in accessing preventive dental services and effective treatments (7). Studies have also indicated that lower-income and educational levels were significantly correlated with severe periodontal disease (8).

The public health significance of periodontitis extends far beyond oral health alone, given its established associations with major non-communicable diseases (NCDs), including diabetes mellitus (9), cardiovascular diseases (10), neurodegenerative disorders (11), and cancers of the head and neck (12). Robust epidemiological evidence consistently identifies severe periodontal disease as a notable risk factor contributing to the onset, progression, and severity of these systemic conditions (7). Specifically, the inflammatory pathways activated in periodontitis are implicated in insulin resistance, atherosclerotic plaque formation, and chronic systemic inflammation, mechanisms directly influencing diabetes control, cardiovascular health, and cognitive decline (9–11). Moreover, periodontitis has been linked with elevated risks for head and neck malignancies through mechanisms involving chronic inflammation and microbial dysbiosis (12). Notably, clinical and population-based research has demonstrated that effective management of periodontal inflammation can lead to measurable improvements in clinical outcomes and prognosis of these associated NCDs (13), underscoring the critical need for integrating periodontal health strategies into global chronic disease prevention and management frameworks.

Despite these well-established associations and the recognized global burden, important knowledge gaps remain regarding inequalities in periodontitis burden across sociodemographic strata. To date, very limited research has specifically investigated sociodemographic disparities in the global burden of periodontal disease. Although one previous study utilizing the Global Burden of Disease (GBD) 2019 dataset assessed inequalities across varying levels of socioeconomic development (14), substantial knowledge gaps remain, particularly regarding recent global epidemiological trends, the dynamics of burden disparities across distinct sociodemographic strata, and projections of future disease trajectories. As it currently stands, this study is the first to comprehensively evaluate global trends in periodontal disease burden using the GBD 2021 dataset aimed to quantify periodontal disease inequalities through advanced socioeconomic indicators such as the Gini index, systematically analyze temporal shifts in these disparities, and employ machine learning techniques to forecast future periodontal disease trajectories under scenarios of unchanged intervention policies. Thus, this research provides evidence to guide targeted global public health responses and policy formulation aimed directly at reducing periodontal disease burden and inequalities, and indirectly at improving the outcomes of NCDs by controlling one of the most important modifiable risk factors.

## 2 Materials and methods

### 2.1 Study design

This study employed a longitudinal epidemiological approach to analyze the global burden of periodontitis, utilizing data from the GBD 2021 study. The dataset, accessed through the Global Health Data Exchange (GHDx) database (https://vizhub.healthdata.org/gbd-results/, accessed on 2 February 2025) (15), provides comprehensive estimates for disease burden metrics across 204 countries and territories. The GBD framework systematically quantifies incidence, prevalence, mortality and disability-adjusted life years (DALYs) to facilitate comparative assessments across diverse population structures and temporal trends from 1990 to 2021 (16).

The burden of periodontitis was assessed using age-standardized prevalence rates (ASPR), age-standardized incidence rates (ASIR), and age-standardized DALYs rates, expressed per 100,000 population to ensure comparability across regions and time periods. Age-standardization was implemented to control for population growth and demographic shifts, ensuring that observed trends reflect true epidemiological patterns rather than artifacts of changing population structures. The inclusion of DALYs as an outcome measure allows for a comprehensive assessment of disease burden by integrating both years of life lost (YLL) due to premature mortality and years lived with disability (YLD), capturing the long-term functional and health-related consequences of periodontitis (17, 18).

Methodological procedures applied in GBD 2021 have been extensively documented in previous literature and validated for comparability across diseases, regions, and time points. The modeling framework incorporates cause-specific mortality estimates, systematic literature reviews, survey data, and health system records, adjusted for heterogeneity in case definitions and reporting biases (19). These methodological refinements ensure that the estimates produced are robust, epidemiologically reliable, and suitable for informing global and national health policies.

By utilizing this comprehensive epidemiological dataset, the present study systematically evaluated the temporal and geographical trends of periodontitis over the past three decades.

### 2.2 Socio-demographic index (SDI)

The Socio-demographic Index (SDI) is a composite metric developed by GBD researchers to quantify developmental status and its association with health outcomes (20). It is derived as the geometric mean of three key determinants: total fertility rate under the age of 25 (TFU25), mean years of education among individuals aged 15 and older (EDU15+), and lag-distributed income (LDI) per capita. The index is scaled from 0 to 1, where 0 represents the lowest level of socio-economic development relevant to health, and 1 reflects the highest possible development level.

SDI exhibits a strong correlation with key health indicators, including mortality rates, life expectancy, and DALYs, making it a robust predictor of disease burden and health disparities across populations. Based on their SDI scores, countries are stratified into five distinct categories:

- Low SDI
- Low-middle SDI
- Middle SDI
- High-middle SDI
- High SDI

This stratification enables a systematic comparison of health outcomes across varying levels of socio-economic development, facilitating the assessment of trends, disparities, and policy-relevant implications in disease burden. SDI data utilized in this study were sourced from the GHDx database, ensuring alignment with the broader GBD analytical framework.

### 2.3 Gini coefficient

To assess the inequality in the global burden of periodontitis, we computed the Gini coefficient for the age-standardized prevalence, incidence, and DALY rates across countries from 1990 to 2021. The Gini coefficient, a measure of statistical dispersion (21), quantifies the degree of inequality in a given distribution, with values ranging from 0 (perfect equality) to 1 (maximum inequality). Higher values indicate greater disparities in disease burden across geographic regions. For each year, we estimated the Gini coefficient, applying country-level values of prevalence, incidence, and DALYs as the outcome variables. The temporal trend of the Gini coefficient was analyzed to identify changes in inequality over time (22).

### 2.4 Statistical methods

A time-series analysis was conducted to examine the longitudinal trends of ASPR, ASIR, and age-standardized DALYs rates for periodontitis from 1990 to 2021. This analysis was performed at the global level and stratified by SDI categories to assess temporal patterns and variations in disease burden across different levels of socio-economic development. The 95% uncertainty intervals (UI) were computed to account for variability in disease estimates over time. The results were visualized using time-series plots, allowing for the identification of overall trends and potential shifts in burden across SDI strata.

To further characterize the distribution of these epidemiological metrics within each SDI category, boxplots were generated. These visualizations provided insight into the spread and variability of periodontitis burden within different development levels, highlighting disparities in disease burden across the five SDI groups. This approach was essential for detecting potential outliers, interquartile range variations, and central tendency differences that might not be immediately apparent from aggregated trend analyses.

A linear regression analysis was subsequently performed to assess the relationship between SDI (modeled as a continuous variable) and each of the three epidemiological measures (ASPR, ASIR, and DALYs). This analysis leveraged the full dataset of 204 countries and territories, covering the entire observation period. The coefficient of determination (*R*^2^) was computed to quantify the proportion of variance in disease burden explained by SDI, thereby evaluating the strength of association between socio-economic development and periodontitis burden. The regression results were visualized using scatterplots with fitted regression lines, which facilitated the identification of linear trends and deviations in the data.

Kruskal–Wallis test was employed to evaluate differences in ASPR, ASIR, and DALYs among the five SDI categories. This non-parametric method was chosen due to its robustness against violations of normality assumptions in continuous data. Where significant differences were detected, Dunn's test for *post-hoc* pairwise comparisons was conducted to determine which SDI groups exhibited statistically significant differences in disease burden.

To further explore temporal shifts in periodontitis burden, Joinpoint regression analysis was applied to detect significant inflection points in the trends of ASPR, ASIR, and DALYs over the 1990–2021 period. The maximum number of joinpoints was set to three, ensuring the identification of major structural changes in trend patterns while maintaining statistical power. To assess the robustness of the Joinpoint regression results and minimize potential overfitting, sensitivity analyses were performed by varying the maximum number of allowed joinpoints. The main trend patterns and inflection points remained consistent across these specifications. Significant changes in rates over time were assessed using the permutation test (23), with *p*-values estimated via Monte Carlo resampling methods to control for type I error inflation (24).

The Annual Percent Change (APC) was calculated for each identified segment to characterize year-over-year fluctuations in periodontitis burden, while the Average Annual Percent Change (AAPC) was computed as a weighted geometric mean of the APCs across all detected segments. APC provides a measure of the relative rate of change in disease burden per year, while AAPC offers a summary measure of long-term trends, adjusting for variations in segment lengths. These metrics enable a detailed temporal characterization of periodontitis burden, identifying periods of accelerated increase, decline, or stability.

All Joinpoint regression analyses were conducted using the Joinpoint Regression Program (Version 5.3.0—November 12, 2024) (25), developed by the National Cancer Institute (Bethesda, MD, USA). All other statistical analyses, including time-series modeling, regression analyses, and non-parametric tests, were performed using Stata IC version 18.0 (26). The time-series plots, boxplots, and scatterplots were generated in Stata, while Joinpoint regression graphs were produced in the Joinpoint Regression Program. A two-tailed *p*-value of less than 0.05 was considered statistically significant for all inferential analyses.

### 2.5 Forecasting and geospatial analysis methods

A time series based XGBoost approach was used (27, 28) with a recursive multi-step strategy, to forecast the ASPR, ASIR and age-standardized DALY values across different SDI categories, along with the confidence intervals (upper and lower bounds). To be able to capture the temporal dependencies better, lag features were generated and introduced into the dataset. To prevent overfitting, we used a two-step approach for the model training. First, we used a timeseries cross-validation with 3 splits, preserving the temporal structure of the data to secure that the training set always precedes the validation set, thus avoiding leakage of future information into training. Second, we performed grid search to fine-tune hyperparameters specifically chosen to reduce model complexity. The number of trees was limited (n estimators: 50, 100), the maximum tree depth restricted (max depth: 2, 3), conservative learning rates selected (learning rate: 0.01, 0.1), and moderate subsampling applied to rows and columns (subsample: 0.8, 1.0; column sample by tree: 0.8, 1.0). The model was trained on the complete historical time series using lagged features to capture temporal dependencies. For out-of-sample forecasting, the last available timepoint served as the initial input, with predicted values recursively fed back to generate multi-step forecasts for a 10-year horizon. The strength of the prediction decreases with each additional forecast step.

To evaluate the predicative accuracy across multiple SDI categories, Relative Root Mean Squared Error (Relative RMSE) was computed for each category. If the Relative RMSE value is less than 1 the model error is small, indicating a good predicative performance, if the value is between 1 and 2 the error is moderate, suggesting an acceptable but not highly accurate prediction, if the value is above 2 the model error is twice the standard deviation of the actual observation, implying poor predicative performance. Out of the six predictions for the SDI categories considering the prevalence, incidence, and DALY separately, two showed moderate predictive performance, while four demonstrated good predictive performance.

For the geospatial representation, the yearly ASPR, ASIR, and DALY values were divided into three equal parts for each country based on available years. The average was then calculated for each of these divisions. A choropleth map was generated, where each country was color-coded according to the average values of prevalence, incidence, and DALY. This process was repeated for each of the three time divisions within prevalence, incidence, and DALY, resulting in a total of nine maps.

Prediction modeling was conducted using the XGBoost library, while geospatial analyses were performed using GeoPandas (29), a Python library for geospatial data handling (30).

## 3 Results

### 3.1 Time-series analysis of periodontitis incidence, prevalence, and DALYs from 1990 to 2021

The global ASPR of periodontitis declined from 12,282 cases per 100,000 population (95% UI: 9,495–15,064) in 1990 to its lowest point in 2010 at 11,377 (95% UI: 9,463–13,183), followed by a gradual increase to 12,498 (95% UI: 10,527–14,493) in 2021.

A similar trend was observed for ASIR, which declined from 1,069 cases per 100,000 population (95% UI: 853–1,240) in 1990 to its lowest recorded value of 1,005 (95% UI: 873–1,136) in 2010, followed by a gradual increase, reaching 1,069 (95% UI: 943–1,205) in 2021, with relative stability in the later years.

**Figure 1:**
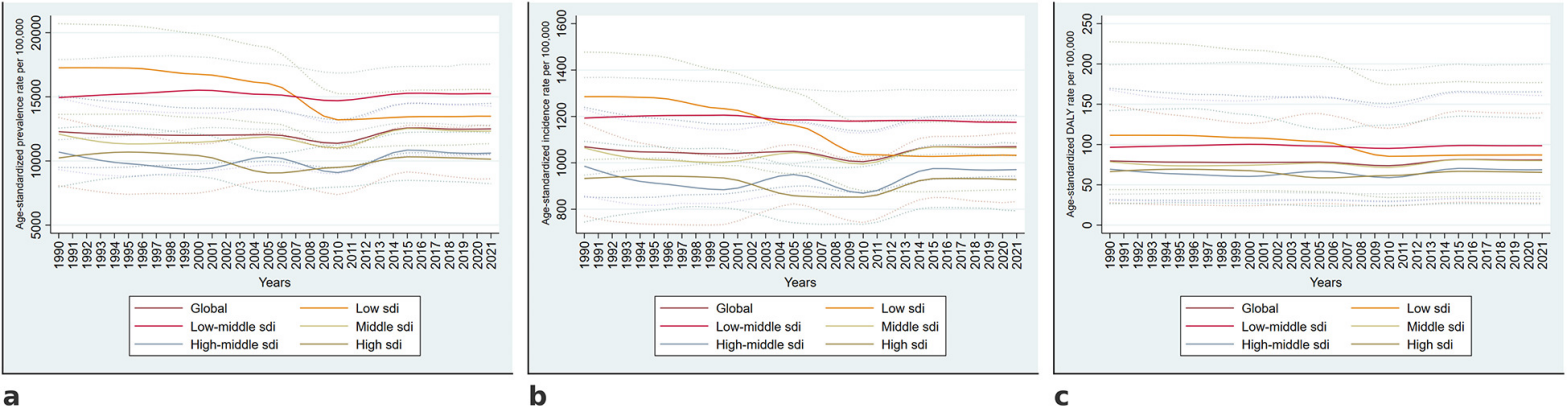
Temporal trends in the burden of periodontitis from 1990 to 2021. Global and SDI-stratified trends in age-standardized prevalence **(a)**, incidence **(b)**, and DALY rates **(c)** per 100,000 population from 1990 to 2021. Solid lines represent estimates, and dotted lines indicate 95% uncertainty intervals.

The global age-standardized DALYs rate for periodontitis declined from 80 per 100,000 population (95% UI: 31–170) in 1990 to its lowest recorded value of 74 (95% UI: 30–151) in 2010, followed by a gradual increase, stabilizing at 81 (95% UI: 32–165) in 2021. (Figures 1a–c).

### 3.2 Distribution of periodontitis burden across socio-dmographic Index categories and statistical comparisons

**Figure 2:**
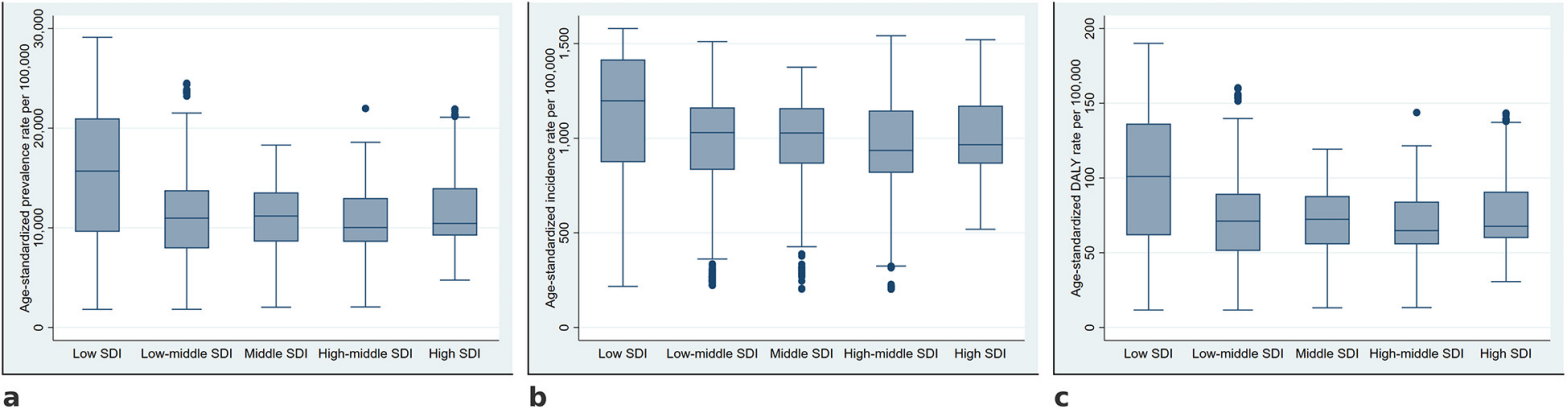
Distribution of age-standardized prevalence, incidence, and DALY rates of periodontitis across socio-demographic Index categories. Boxplots illustrate the distribution of age-standardized prevalence **(a)**, incidence **(b)**, and DALY rates **(c)** of periodontitis across the five SDI categories.

Age-standardized prevalence, incidence, and DALY rates demonstrated significant variability across SDI categories depicted in Figures 2a–c. The highest median prevalence was observed in low SDI countries, with progressively decreasing values toward high SDI regions. Incidence rates followed a similar pattern, with low SDI countries exhibiting the highest values, while high-middle and high SDI categories had the lowest. The distribution of DALY rates showed a clear gradient, with lower SDI categories having greater disease burden.

The Kruskal–Wallis test results, shown in Table 1 indicated a statistically significant difference in the ASPR across SDI categories (*p* < 0.001). Dunn's *post-hoc* comparisons revealed significantly higher prevalence in low SDI countries compared to all other categories (all *p* < 0.001), with the largest mean rank difference observed between low SDI and high-middle SDI (19.69). The variations in ASIR across SDI categories were also statistically significant (*p* < 0.001). Dunn's *post-hoc* analysis indicated that low SDI countries exhibited the highest ASIR, significantly exceeding all other SDI categories, with the largest mean rank difference observed when compared to high-middle SDI countries (16.73, *p* < 0.001). The Kruskal–Wallis test confirmed significant differences in age-standardized DALY rates across SDI categories (*p* < 0.001). The highest burden was observed in low SDI countries, with significantly higher DALY rates than all other SDI categories (rank differences: 18.91 vs. low-middle SDI, 16.39 vs. middle SDI, 19.56 vs. high-middle SDI, and 10.41 vs. high SDI; all *p* < 0.001) (Table 1).

**Table 1:** Comparative analysis of periodontitis burden across socio-demographic index categories using the Kruskal–Wallis and Dunn's *post hoc* tests.

Metric	K–Wallis *p*-value	Pairwise comparison	Mean rank difference	Adj. Sig
Prevalence	*p* < 0.001	Low SDI vs. Low-middle SDI	19.12	***p* < 0.001**
		Low SDI vs. Middle SDI	16.57	***p* < 0.001**
		Low SDI vs. High-middle SDI	19.69	***p* < 0.001**
		Low SDI vs. High SDI	10.6	***p* < 0.001**
		Low-middle SDI vs. Middle SDI	−0.5	0.3103
		Low-middle SDI vs. High-middle SDI	2.72	**0.003**
		Low-middle SDI vs. High SDI	−3.29	***p* < 0.001**
		Middle SDI vs. High-middle SDI	2.95	**0.002**
		Middle SDI vs. High SDI	−2.71	**0.003**
		High-middle SDI vs. High SDI	−5.19	***p* < 0.001**
Incidence	*p* < 0.001	Low SDI vs. Low-middle SDI	14.58	***p* < 0.001**
		Low SDI vs. Middle SDI	12.46	***p* < 0.001**
		Low SDI vs. High-middle SDI	16.73	***p* < 0.001**
		Low SDI vs. High SDI	8.8	***p* < 0.001**
		Low-middle SDI vs. Middle SDI	−0.54	0.294
		Low-middle SDI vs. High-middle SDI	3.77	***p* < 0.001**
		Low-middle SDI vs. High SDI	−1.79	**0.037**
		Middle SDI vs. High-middle SDI	3.95	***p* < 0.001**
		Middle SDI vs. High SDI	−1.26	0.103
		High-middle SDI vs. High SDI	−4.59	***p* < 0.001**
DALY	*p* < 0.001	Low SDI vs. Low-middle SDI	18.91	***p* < 0.001**
		Low SDI vs. Middle SDI	16.39	***p* < 0.001**
		Low SDI vs. High-middle SDI	19.56	***p* < 0.001**
		Low SDI vs. High SDI	10.41	***p* < 0.001**
		Low-middle SDI vs. Middle SDI	−0.49	0.311
		Low-middle SDI vs. High-middle SDI	2.78	**0.003**
		Low-middle SDI vs. High SDI	−3.32	***p* < 0.001**
		Middle SDI vs. High-middle SDI	2.99	**0.001**
		Middle SDI vs. High SDI	−2.74	**0.003**
		High-middle SDI vs. High SDI	−5.26	***p* < 0.001**

Bold values indicate statistical significance (*p* < 0.05). K–Wallis, Kruskal–Wallis test; Adj. Sig, adjusted significance. SDI, socio-demographic index; DALY, disability-adjusted life years.

**Figure 3:**
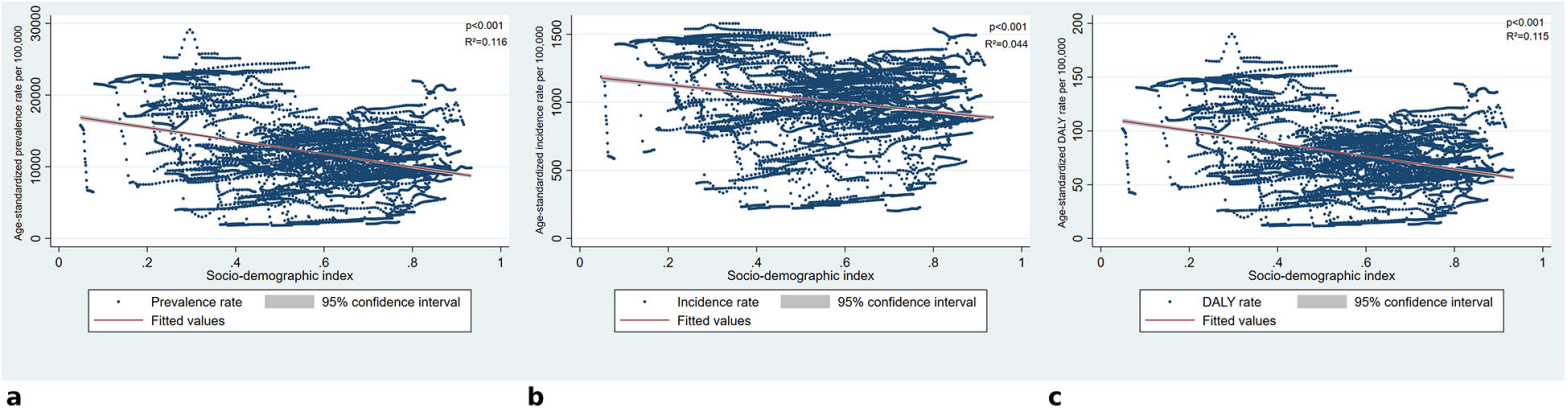
Association between socio-demographic Index and periodontitis burden. **(a)** Presents the relationship between SDI and age-standardized prevalence rates, **(b)** shows SDI against age-standardized incidence rates, and Panel c illustrates SDI vs. age-standardized DALY rates. Linear regression lines indicate a negative association across all measures, with statistically significant results (*p* < 0.001). R-squared values suggest that SDI accounts for a limited proportion of the variance, with the strongest association observed for prevalence (*R*^2^ = 0.116) and DALYs (*R*^2^ = 0.115), while incidence had the lowest explanatory power (*R*^2^ = 0.044).

The linear regression model assessing the association between SDI and the age-standardized prevalence rate of periodontitis was statistically significant (F = 859.75, *p* < 0.001). The model explained 11.6% of the variance (*R*^2^ = 0.116). SDI was negatively associated with prevalence (*β* = −9,181.24, 95% CI: −9,795.06 to −8,567.41, *p* < 0.001), indicating a decreasing trend in prevalence with increasing SDI levels. The correlation between SDI and ASIR was also statistically significant (F = 299.31, *p* < 0.001). The model explained 4.4% of the variance (*R*^2^ = 0.044). SDI demonstrated a negative association with incidence (*β* = −327.99, 95% CI: −365.15 to −290.82, *p* < 0.001), indicating a decreasing trend in incidence rates as SDI levels increased. The SDI was also significantly correlated with the age-standardized DALYs rates (F = 845.04, *p* < 0.001). The model accounted for 11.5% of the variance (*R*^2^ = 0.115). SDI exhibited a negative association with DALY rates (*β* = −59.50, 95% CI: −63.51 to −55.49, *p* < 0.001), indicating a decline in disease burden with increasing SDI (Figures 3a–c).

### 3.3 Joinpoint regression analysis of disease burden by SDI

At the global level, the ASPR of periodontitis showed a declining trend from 1990 to 2010 (1990–2006: −0.121%; *p* < 0.05, 2006–2010: −1.313%; *p* < 0.05), followed by a significant increase (2010–2015: 2.132%; *p* < 0.05), before stabilizing. The AAPC (1990–2021) remained negative in low (−0.792%; *p* < 0.05) and high SDI (−0.081%; *p* < 0.05), while low-middle (0.064%; *p* < 0.05), middle (0.158%; *p* < 0.05), and global (0.075%; *p* < 0.05) trends showed a slight overall increase.

The global ASIR declined significantly between 1990 and 2006 (APC: −0.123%, *p* < 0.05) and further decreased from 2006 to 2010 (APC: −0.886%, *p* < 0.05), followed by a significant rise from 2010 to 2015 (APC: 1.306%, *p* < 0.05) before stabilizing after 2015. Low SDI countries experienced the most pronounced decline from 1996 to 2006 (APC: −1.053%, *p* < 0.05) maintaining an overall downward trend (AAPC: −0.719%, *p* < 0.05).

**Figure 4:**
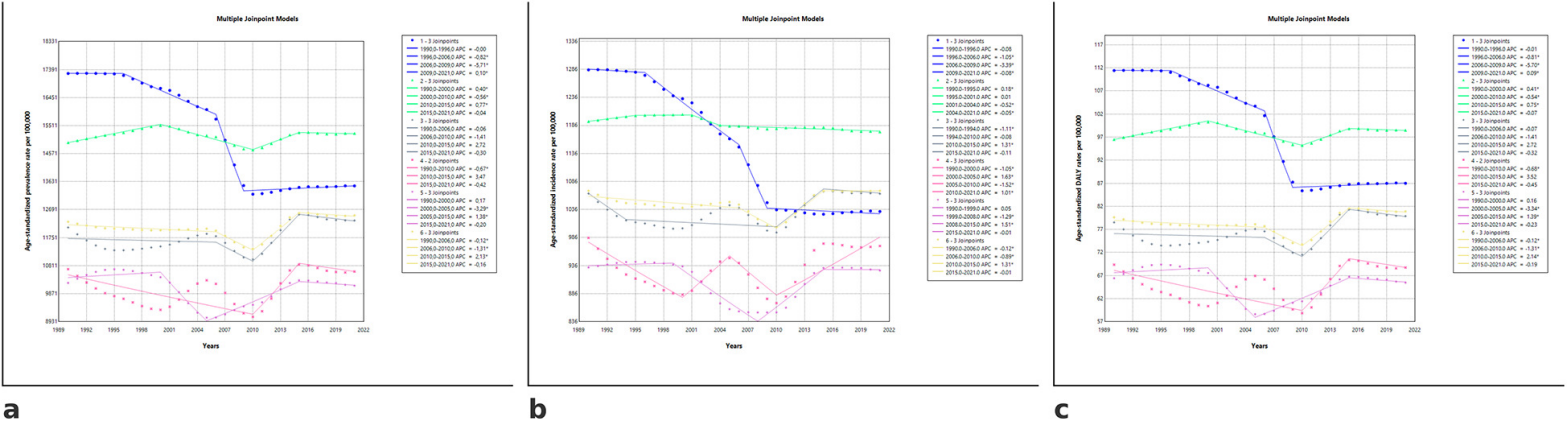
Joinpoint regression models for age-standardized prevalence, incidence, and DALY rates of periodontitis (1990–2021) across SDI categories. The figures illustrate joinpoint regression trends in the age-standardized prevalence **(a)**, incidence **(b)**, and DALY rates **(c)** of periodontitis from 1990 to 2021 across different SDI categories. Each segment represents a distinct trend period, with corresponding Annual Percentage Change (APC) values. Asterisks (*) indicate statistically significant trends (*p* < 0.05). SDI categories are coded as follows in the software: 1 = Low SDI, 2 = Low-middle SDI, 3 = Middle SDI, 4 = High-middle SDI, 5 = High SDI, and 6 = Global.

Age-standardized DALY rates globally declined significantly from 1990 to 2006 (APC: −0.123%, *p* < 0.05), followed by an accelerated reduction between 2006 and 2010 (APC: −1.308%, *p* < 0.05). A subsequent increase from 2010 to 2015 (APC: 2.139%, *p* < 0.05) was followed by stabilization after 2015, with an overall AAPC of 0.072% (*p* < 0.05). Low SDI regions showed the sharpest decline from 1996 to 2006 (APC: −0.814%, *p* < 0.05), with a steeper drop between 2006 and 2009 (APC: −5.700%, *p* < 0.05), before transitioning into a slight increasing trend (APC: 0.091%, *p* < 0.05, 2009–2021), resulting in an overall AAPC of −0.794% (*p* < 0.05). The full Joinpoint regression output is shown in Table 2 and visualized in Figures 4a–c. (Table 2).

**Table 2:** Joinpoint regression analysis of age-standardized prevalence, incidence, and DALY rates of periodontitis (1990–2021) across socio-demographic Index levels and globally.

Prevalence	Trend 1		Trend 2		Trend 3		Trend 4		1990-2021
	Period	APC (%)	Period	APC (%)	Period	APC (%)	Period	APC (%)	AAPC (%)
Low SDI	1990–1996	−0.004	1996–2006	−0.822*	2006–2009	−5.705*	2009–2021	0.104*	−0.792*
Low-middle SDI	1990–2000	0.404*	2000–2010	−0.560*	2010–2015	0.768*	2015–2021	−0.045	0.064*
Middle SDI	1990–2006	−0.065	2006–2010	−1.412	2010–2015	2.72	2015–2021	−0.298	0.158*
High-middle SDI	1990–2010	−0.672*	2010–2015	3.475	2015–2021	−0.424	–	–	0.034
High SDI	1990–2000	0.172	2000–2005	−3.292*	2005–2015	1.383*	2015–2021	−0.203	−0.081*
Global	1990–2006	−0.121*	2006–2010	−1.313*	2010–2015	2.132*	2015–2021	−0.165	0.075*
Incidence	Trend 1		Trend 2		Trend 3		Trend 4		1990–2021
	Period	APC (%)	Period	APC (%)	Period	APC (%)	Period	APC (%)	AAPC (%)
Low SDI	1990–1996	−0.075	1996–2006	−1.053*	2006–2009	−3.395*	2009–2021	−0.080*	−0.719*
Low-middle SDI	1990–1995	0.178*	1995–2001	0.009	2001–2004	−0.523*	2004–2021	−0.050*	−0.047*
Middle SDI	1990–1994	−1.112*	1994–2010	−0.079	2010–2015	1.306*	2015–2021	−0.115	0.002
High-middle SDI	1990–2000	−1.055*	2000–2005	1.630*	2005–2010	−1.516*	2010–2021	1.013*	0.03
High SDI	1990–1999	0.054	1999–2008	−1.291*	2008–2015	1.507*	2015–2021	−0.006	−0.025
Global	1990–2006	−0.123*	2006–2010	−0.886*	2010–2015	1.306*	2015–2021	−0.009	0.029
DALY	Trend 1		Trend 2		Trend 3		Trend 4		1990–2021
	Period	APC (%)	Period	APC (%)	Period	APC (%)	Period	APC (%)	AAPC (%)
Low SDI	1990–1996	−0.005	1996–2006	−0.814*	2006–2009	−5.700*	2009–2021	0.091*	−0.794*
Low-middle SDI	1990–2000	0.408*	2000–2010	−0.539*	2010–2015	0.751*	2015–2021	−0.069	0.065*
Middle SDI	1990–2006	−0.068	2006–2010	−1.409	2010–2015	2.725	2015–2021	−0.32	0.154*
High-middle SDI	1990–2010	−0.681*	2010–2015	3.524	2015–2021	−0.455			0.071
High SDI	1990–2000	0.159	2000–2005	−3.343*	2005–2015	1.388*	2015–2021	−0.234	−0.098*
Global	1990–2006	−0.123*	2006–2010	−1.308*	2010–2015	2.139*	2015–2021	−0.188	0.072*

The table summarizes joinpoint regression analysis of age-standardized prevalence, incidence, and DALY rates of periodontitis (1990–2021) across SDI categories. APC reflects trend changes within specific periods, while AAPC represents the overall trend. Statistically significant values are indicated by an asterisk (*). SDI, socio-demographic index; APC, annual percentage change; AAPC, average annual percentage change.

The global distribution of periodontitis prevalence exhibited changing geographic patterns over time. Between 1990 and 2000, the highest prevalence rates were observed in sub-Saharan Africa, parts of the Middle East, and South Asia. In the most recent period (2012–2021), the global prevalence trends showed a modest overall decline, particularly in high-income regions, while persistently high burden remained in lower-income countries.

Regarding age-standardized incidence rate, a clear regional pattern emerges, with persistently high incidence rates in sub-Saharan Africa and parts of South Asia, throughout the three decades. Countries in Western and Northern Europe, North America, and Australia consistently exhibit lower incidence rates, suggesting better periodontal health outcomes and disease prevention strategies in high-income regions.

**Figure 5:**
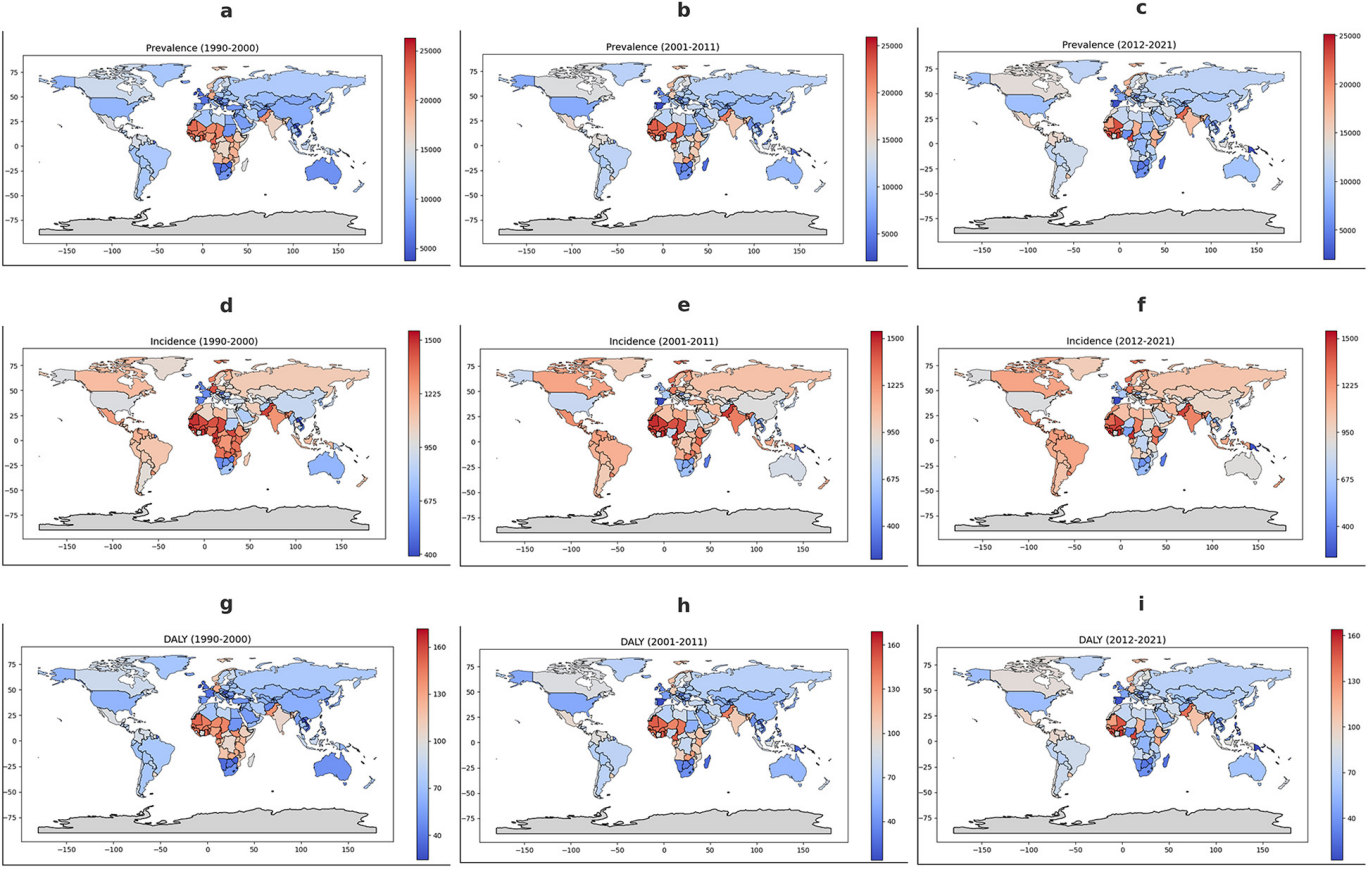
Geographical distribution of periodontitis burden from 1990 to 2021. The figure presents the geographical distribution of periodontitis burden from 1990 to 2021. **(a–c)** Show the average age-standardized prevalence rates per 100,000 for 1990–2000, 2001–2011, and 2012–2021, respectively. **(d–f)** Depict incidence rates per 100,000 for the same periods, while **(g–i)** illustrate disability-adjusted life years (DALY) per 100,000. Color gradients indicate burden levels, with red shades representing higher rates and blue shades indicating lower rates, as shown in the corresponding scale bars. Gray areas denote missing or unavailable data.

The analysis of DALY rates for periodontitis from 1990 to 2021 reveals persistent global disparities, with Sub-Saharan Africa and parts of South Asia consistently exhibit the highest burden, with DALY rates exceeding 160 per 100,000 (Figures 5a–i).

### 3.4 Global health inequality in periodontitis burden

**Figure 6:**
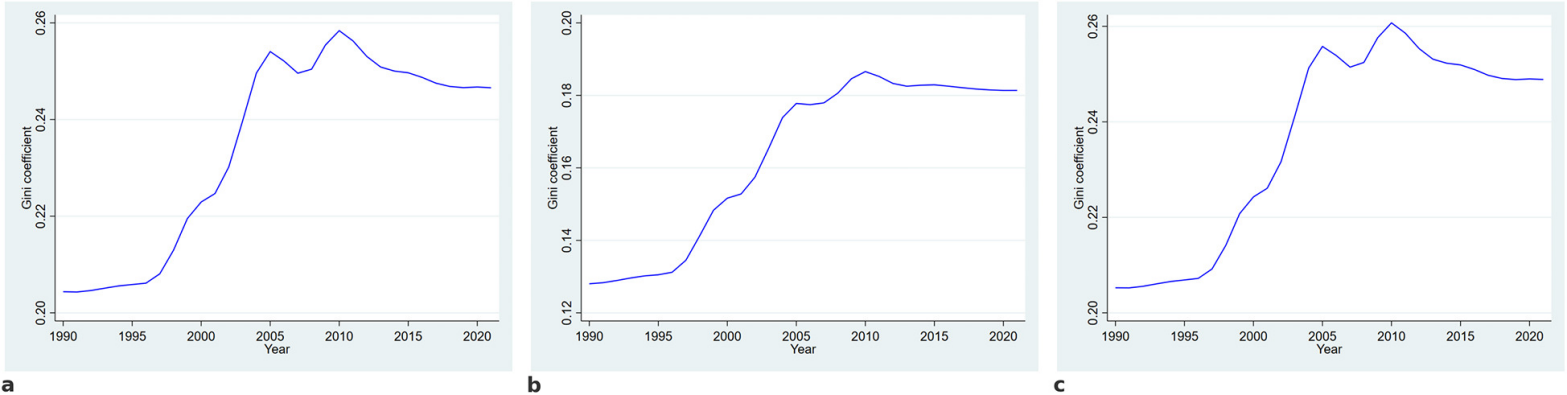
Trends in the gini coefficient for age-standardized prevalence **(a)**, incidence **(b)**, and disability-adjusted life years **(c)** of periodontitis from 1990 to 2021. The Gini coefficient was computed to assess global inequalities in periodontitis burden across countries. A higher Gini coefficient indicates greater disparity.

The Gini coefficient for ASPR exhibited a marked increase from approximately 0.21 in 1990 to a peak of 0.26 around 2010, indicating a widening disparity in disease burden across countries during this period. After reaching its peak, the coefficient demonstrated a gradual decrease, declining to about 0.24 by 2021, and remained consistently above pre-2000 levels throughout the subsequent years. A similar pattern was observed for ASIR, with the Gini coefficient rising from approximately 0.13 in 1990 to 0.19 in 2010, followed by a slight decline and stabilization at around 0.18 in the most recent years. For DALY rates, the Gini coefficient increased from roughly 0.20 in 1990 to a maximum of 0.26 in the mid-2000s, after which it declined moderately and stabilized at approximately 0.24 through 2021. These data indicate that, while disparities in periodontitis burden increased markedly during the first two decades, there was a subsequent period of slight reduction and stabilization in the level of global inequality after 2010 (Figures 6a–c).

### 3.5 Predicted trends in periodontitis burden across SDI categories

**Figure 7:**
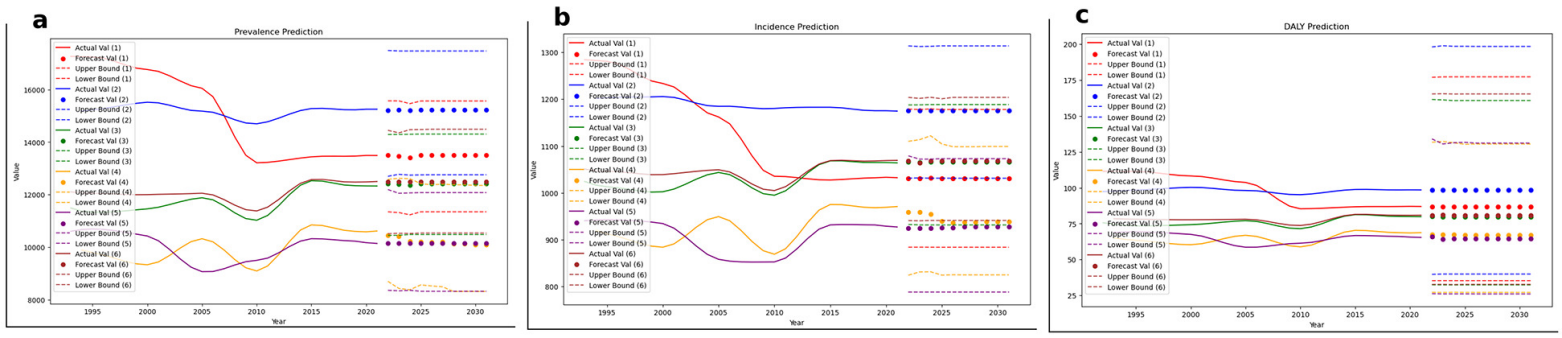
Projected trends in age-standardized prevalence, incidence, and DALY rates of periodontitis until 2030 across SDI categories. **(a–c)** depict projections for prevalence, incidence, and DALY rates, respectively, modeled until 2030. Solid lines represent historical trends, while dots indicate forecasted values. Dotted lines denote upper and lower confidence bounds. SDI categories are coded as follows: 1 = Low SDI, 2 = Low-middle SDI, 3 = Middle SDI, 4 = High-middle SDI, 5 = High SDI, 6 = Global estimate.

Before interpreting these projections, it is important to note that model performance varied by indicator and SDI group: out of the six predictions (prevalence, incidence, and DALY across SDI categories), two demonstrated moderate predictive performance while four showed good predictive performance. Trend projectionspresented in Figures 7a–c reveal a persistent SDI gradient, with low-SDI regions maintaining the highest burden across all indicators and showing minimal expected reductions over time. In contrast, high-middle and high-SDI regions exhibit stabilized trends at lower levels. Low-middle SDI regions display slight fluctuations, with projected trends suggesting either stability or marginal declines. Global projections suggest that overall reductions will be modest and primarily driven by improvements in higher SDI categories. Incidence and DALY projections follow a similar gradient, with low-SDI regions remain disproportionately affected with no substantial decline expected, while higher SDI categories are forecasted to maintain a lower and stable disease burden. Notably, confidence intervals indicate greater uncertainty in low-SDI projections, highlighting potential variability in future trends (Figures 7a–c).

## 4 Discussion

The current study aimed to assess the inequalities in the burden of periodontal disease between different sociodemographic development levels. Globally, the burden of periodontitis remained persistently high from 1990 to 2021, with disparities across SDI categories. Prevalence rates demonstrated relative stability at the global level, yet trends diverged across SDI strata, with low and low-middle SDI countries experiencing significant increases, while high SDI regions saw gradual declines or stabilization. Joinpoint analysis revealed periods of accelerated burden reduction in high SDI settings, particularly post-2010, whereas low SDI regions experienced consistent increases, reflecting growing inequities. Incidence rates followed a similar pattern, with the largest sustained decreases observed in high SDI countries, while low and low-middle SDI countries exhibited slower declines or modest increases, underscoring persistent challenges in preventive care access. DALY trends further emphasized the disproportionate impact on lower SDI countries, reinforcing the cumulative burden of periodontitis-related disability in resource-constrained regions. Inequality metrics corroborated these findings, with Gini coefficients indicating widening disparities, particularly between high and low SDI settings. Predictive modeling suggested that, without intervention, periodontitis prevalence and DALY rates will continue increasing in low SDI regions, while high SDI countries are projected to maintain current levels or improve slightly.

Periodontal diseases disproportionately burden populations in developing countries compared to those residing in developed nations, a disparity extensively documented since the foundational epidemiological work by Ramfjord et al. (31). This early seminal study highlighted the elevated prevalence and severity of periodontal conditions in resource-constrained regions, a phenomenon attributed primarily to socioeconomic determinants and limited accessibility to adequate oral healthcare services. Consistent with these observations, subsequent research further outlined disparities along racial and socio-demographic lines, focusing on the interactions between socioeconomic factors, oral hygiene behaviors, healthcare access, and microbiological determinants in shaping periodontal disease outcomes.

For instance, in an influential population-based investigation among 690 older adults dentate individuals (aged ≥65 years) residing in five counties across North Carolina, significant racial disparities were observed (32). The study, employing clinical periodontal assessments (including pocket depth and gingival recession measurements), identified a markedly elevated prevalence of severe periodontal disease among Black participants compared to their White counterparts (32). Specifically, a “serious periodontal condition” was rigorously defined by the presence of four or more sites with clinical attachment loss exceeding 5 mm, accompanied by at least one periodontal pocket exceeding 4 mm in depth. Multivariate logistic regression analyses underscored distinct risk profiles: among Black participants, risk factors included tobacco use, infrequent dental care utilization, bleeding gums, and the presence of periodontal pathogens such as Bacteroides gingivalis. Similarly, for White participants, tobacco consumption emerged as the predominant risk factor, followed by infrequent dental visits and pathogen colonization (32). These findings draw a globally accepted picture of how periodontal health disparities are intricately linked to broader socio-environmental and behavioral determinants. Studying these determinants, and their links with the changing burden of periodontal disease can help mitigate disparities and inform general public health policies to tailor interventions and policy changes based on these cultural differences and based on the uniqueness of each affected region.

In the present analysis, low SDI countries, exemplified by nations such as Chad, Mozambique, Afghanistan, and Ethiopia, demonstrated marked increases in periodontal disease ASPR, ASIR and DALY between 1990 and 2021. Joinpoint regression analysis indicated significant fluctuations, characterized by brief intervals of improvement overshadowed by sustained periods of increasing disease burden. For instance, the 2006–2009 period witnessed sharp annual declines in prevalence (−5.70%) and incidence (−3.40%), suggesting transient impacts possibly attributable to data reporting or case reporting methodology. However, these gains proved unsustainable, as subsequent years saw renewed increases in periodontal disease measures. This oscillation points towards the profound challenges low SDI countries face in maintaining progress amid systemic health resource limitations.

The structural determinants underlying this heavy burden were highlighted in recent studies conducted in Ethiopia and across a broader set of 27 low-income countries. Gizaw et al. described substantial deficits in basic oral hygiene practices among rural Ethiopian communities (33), with only about 30% of households regularly practicing tooth brushing, typically using rudimentary methods such as chewing sticks. Alarmingly, gum pricking, which is a harmful cultural practice involving deliberate trauma to gingival tissues,was reported by approximately 15% of households, underscoring deep-seated misconceptions and a lack of preventive oral health knowledge. Notably, the provision of oral hygiene education markedly improved these practices (Adjusted Odds Ratio: 1.66, 95% CI: 1.26–2.21), yet widespread implementation of such educational interventions remains limited due to infrastructural and financial constraints (33).

Complementing these behavioral determinants, structural deficiencies within healthcare systems further exacerbate periodontal disease disparities in low SDI contexts. A comprehensive scoping review by Luan et al. examining oral health coverage in 27 low-income nations found minimal political and financial investment toward oral health (34), resulting in extremely low utilization rates and virtually no preventive or periodontal-focused public health interventions. Predominantly, oral health services were limited to symptomatic management, such as tooth extractions, due to severe shortages of dental professionals—only 0.51 dentists per 10,000 population compared to 2.83 and 7.62 in middle- and high-income countries, respectively (34). These profound resource limitations hinder periodontal care, amplifying inequalities and perpetuating a cycle of chronic oral diseases and associated systemic health consequences.

Such structural and behavioral realities directly contribute to the findings from our inequality assessments. The Gini coefficient analysis presented herein underscored increasing global inequalities, with disease burden disproportionately concentrated in low SDI countries. Notably, after 2010, the Gini coefficients for periodontitis prevalence, incidence, and DALY rates demonstrated a modest decline and subsequent stabilization, following the pronounced increases observed in the preceding decades. This recent trend may be partially explained by a combination of factors, including the gradual implementation of targeted oral health interventions, increased international focus on reducing health disparities, and improvements in disease surveillance and reporting practices (31). Additionally, successful public health initiatives in certain regions and advancements in preventive care coverage may have contributed to narrowing inequalities in the global burden of periodontitis during this period (34).

These analytical findings resonate with prior research identifying race, ethnicity, socioeconomic status, and access to healthcare as significant determinants of periodontal disease outcomes. Beck et al. for instance, demonstrated substantial racial disparities in periodontal disease severity among older adults populations (32), highlighting the complex interaction of socioeconomic disadvantage, healthcare utilization behaviors, and microbial exposures. Similarly, in low SDI countries, socioeconomic barriers profoundly influence periodontal disease outcomes through restricted access to preventive education, professional dental care, and effective treatment modalities.

The low-middle SDI quintile, represented by Egypt due to its large population size and key location in both the mediterranean as a meeting point between the middle eastern and north African regions, consistently showed the highest periodontal disease burden. This observation aligns with existing literature from Egypt, where national surveys have consistently reported significant disparities in periodontal disease prevalence driven by sociodemographic determinants such as education, smoking, and residency (35). Our analyses demonstrated persistently high prevalence and DALYs rates within this SDI stratum, coupled with modest reductions in incidence rates from 1990 to 2021, potentially attributed to limited access to preventive care and resources predominantly focused on symptomatic relief rather than preventive interventions (36). The notable geographical heterogeneity, depicted clearly through the maps and reinforced by the Lorenz curve and Gini coefficient analyses, highlights substantial inequalities in periodontal disease burden distribution. Specifically, the Gini coefficients for incidence and DALYs in low-middle SDI countries revealed rising inequality, suggesting persistent disparities despite global improvements. These findings are consistent with regional studies from Africa and the Middle East, where disparities in oral health outcomes have been primarily attributed to structural issues such as limited healthcare infrastructure, inadequate preventive measures, and socioeconomic barriers (37, 38). Consequently, addressing periodontal health in these regions necessitates a paradigm shift from traditional, individualized oral healthcare strategies to comprehensive public health approaches targeting social determinants of health, systemic inequities, and common risk factors shared with other NCDs (37).

The epidemiological findings observed in middle SDI countries, represented in this context by Brazil, align with existing literature highlighting substantial regional disparities and social inequalities in periodontal disease prevalence. Consistent with prior comprehensive national surveys, such as SB Brasil 2003 and 2010, Brazil exhibits notable differences in periodontal health indicators across regions, reflecting variations in socioeconomic conditions, access to dental care, and healthcare infrastructure (39). Specifically, periodontitis prevalence remains significantly high in Brazil, affecting over half of the adult population, a figure notably elevated compared to high-SDI nations (39). This situation underscores persistent gaps in dental care coverage, exacerbated by limited availability and unequal distribution of dental professionals and specialized centers, especially pronounced in less-developed regions such as the North and Northeast (40, 41). The literature further emphasizes the impact of socioeconomic factors, including education and income, in determining periodontal health outcomes, echoing our analysis's identification of the SDI as a determinant factor. Additionally, methodological limitations in existing studies, primarily due to the reliance on partial-mouth periodontal examinations such as the Community Periodontal Index (39), suggest that true prevalence may even exceed current estimations. Thus, despite Brazil's progressive oral health policies like ‘Brasil Sorridente,’ considerable efforts remain necessary to mitigate regional inequalities, improve comprehensive periodontal assessments, and ensure equitable dental service access to accurately measure and effectively reduce the burden of periodontal diseases in middle-SDI contexts. While Brazilian studies demonstrated intra-country inequalities, our study extends this to inter-country comparisons, showing that middle SDI nations collectively experience sustained periodontitis burden, likely due to similar socioeconomic structures and healthcare inefficiencies.

The three aforementioned SDI categories, namely low SDI, low-middle SDI and middle SDI are particularly exposed as well to the harms of tobacco use. Tobacco is a strong predictor of periodontal disease (42), low and middle SDI countries were and still are targets for the tobacco industry (43). In these countries in particular there is a rising need to implement tobacco control policies, such as stricter control for selling tobacco products to underage individuals, increasing taxation on these products, and banning their use in areas of education and healthcare.

Our analysis, aligned with previous findings from the Global Burden of Disease studies by Chen et al. and Wu et al. (14, 44), demonstrates a pronounced negative association between the SDI and the burden of severe periodontitis, particularly in high-middle and high SDI countries. This negative association is evident through consistently lower age-standardized rates of prevalence, incidence, and DALYs observed in higher SDI regions compared to their lower counterparts. However, despite this overarching trend, our study identified notable fluctuations in both prevalence and incidence within high-middle and high SDI settings over recent decades. Specifically, these regions experienced periods of decline in periodontal disease burden interspersed with episodes of stabilization or modest increases, which may reflect underlying socioeconomic disparities and persistent inequalities in healthcare accessibility and utilization. This interpretation is further supported by the findings of Wang et al. (45), highlighting that even within affluent countries, marginalized populations encounter significant financial barriers to oral healthcare, thus perpetuating disparities in periodontal health outcomes.

In high-middle and high-income countries, observed trends in periodontal disease burden reflect the combined impact of policies enhancing dental care access, preventive care initiatives, integrated screening programs, and targeted interventions against common risk factors such as tobacco, diabetes, and alcohol consumption. For example, European countries like Norway and Germany have introduced public insurance coverage specifically for periodontal treatment, particularly benefiting individuals with diabetes, thereby reducing financial barriers and facilitating timely periodontal care (46). Similarly, the UK's integration of periodontal screening into primary care for diabetic patients exemplifies a successful cross-disciplinary approach (47). North American countries, particularly the United States and Canada, have achieved significant oral health gains through community fluoridation programs, rigorous tobacco control measures—including taxation, advertising bans, and public education—and policies facilitating periodontal treatment coverage for at-risk populations (48, 49). In East Asia, countries such as Japan and Taiwan have demonstrated substantial reductions in periodontal disease burden by adopting comprehensive preventive strategies like Japan's widely acclaimed “8020 Campaign” and Taiwan's universal dental coverage that promotes regular preventive visits (50). Additionally, targeted tobacco control policies, notably South Korea's significant cigarette tax increase in 2015, have substantially lowered smoking prevalence, indirectly benefiting periodontal health (46).

Future projections indicate that the periodontal disease burden will remain substantially higher in lower SDI groups compared to higher SDI groups, emphasizing that, despite anticipated stabilization or modest declines in high and high-middle SDI regions, marked disparities associated with socioeconomic development are expected to persist. As indicated by our predictive models, countries with lower socioeconomic positions or higher within-country inequalities may not benefit equally from existing preventive and treatment interventions. This underscores the urgent necessity to specifically target vulnerable and socioeconomically disadvantaged groups through inclusive policies such as expanding equitable access to dental care, tailored preventive interventions, and integrating oral health screening into primary care, especially for high-risk populations. Without actively addressing these structural inequalities, future progress in periodontal disease management may disproportionately favor affluent populations, further amplifying existing disparities and undermining overall population health gains. It is noteworthy that the machine learning-based projections for most SDI categories revealed relatively stable trends over the forecast period, closely reflecting the observed values in the most recent years. This pattern likely arises from the plateauing of epidemiological trends in the underlying data, which is faithfully reproduced by the XGBoost model, as expected in robust time-series forecasting. The pronounced drop in the low SDI category between 2005 and 2010, not mirrored in the low-middle SDI category, may reflect shifts in data quality, changes in regional health policy, or potential artifacts in the GBD database for this period. Such divergent trends warrant further investigation to determine whether they represent true epidemiological change, reporting improvements, or other external factors. While the projected trends may appear “flat,” this is consistent with a lack of major change in underlying determinants during the training period, rather than a limitation of the machine learning approach itself.

Low-cost community oral health interventions have demonstrated positive impacts on oral health outcomes and are often feasible at scale. For example, community water fluoridation is a proven, cost-effective measure that significantly reduces dental caries prevalence and can even narrow oral health disparities in populations where it is implemented (51). Similarly, school-based oral hygiene programs, including supervised toothbrushing and health education, have been shown to improve children's oral hygiene and reduce caries incidence in low-resource settings (52). Community-wide oral health education initiatives also increase oral health knowledge and encourage healthier behaviors, especially when combined with other preventive measures, as evidenced by rural outreach programs that improved oral hygiene practices and dental attendance (53). Moreover, integrating basic oral healthcare into primary care services (for instance, training community health workers and primary care providers to deliver preventive dental advice and referrals) has been identified as a sustainable strategy to expand access to oral care in underserved communities (54). Collectively, these interventions are effective and scalable approaches for improving oral health and reducing the burden of oral diseases in resource-limited settings.

A key strength of this study is the comprehensive population-based analysis of global patterns and trends in periodontal disease burden across a lengthy period and diverse socioeconomic contexts, using standardized and integrated data from the Global Burden of Disease Study. This facilitates a robust assessment of international disparities and the evaluation of temporal trends, offering valuable insights for public health policy and resource allocation. However, the study is subject to certain limitations inherent to the GBD methodology. The joinpoint regression analysis results could vary depending on parameter settings and data availability, potentially influencing observed trends. Aggregating data at national levels may conceal within-country variations and socioeconomic inequalities in periodontal health. Variability in diagnostic criteria, data collection methods, ethnicity, lifestyle factors, and healthcare access across countries further complicates data consistency and comparability. Additionally, the reliance on modelled estimates in regions with limited primary data may result in underestimations of periodontal disease burden, particularly in lower-resource settings. The aggregated nature of the data also restricts analysis of individual clinical factors such as disease severity, oral hygiene habits, and specific treatment outcomes. Moreover, the absence of individual-level data on unique behavioral risk factors, such as gum piercing or other culturally specific practices, precluded the assessment of their potential confounding effects on the observed associations. Despite these limitations, the methodology and databases employed effectively identify important global trends and highlight socioeconomic inequalities in periodontal disease burden.

## 5 Conclusion

The global burden of periodontal diseases remains substantial, with a disproportionate impact on low and middle-SDI regions. In contrast, trends in high-SDI countries were generally stable or declining, although some variability was observed across different periods and indicators. This highlights the existence of significant socio-economic inequalities in periodontal health. In order to address this public health challenge in a sustainable manner, there is an urgent need for tailored strategies with a focus on ensuring equitable oral healthcare provision, the strengthening of preventive measures such as tobacco control, and the integration of oral health into broader primary healthcare frameworks. Further research must prioritize evaluating policy effectiveness, exploring context-specific barriers to care, and developing interventions to mitigate the growing disparities in periodontal disease burden worldwide.

## Data Availability

Publicly available datasets were analyzed in this study. This data can be found here: https://vizhub.healthdata.org/gbd-results/.
